# Induction of Type I and Type III Interferons by *Borrelia burgdorferi* Correlates with Pathogenesis and Requires Linear Plasmid 36

**DOI:** 10.1371/journal.pone.0100174

**Published:** 2014-06-19

**Authors:** Michelle A. Krupna-Gaylord, Dionysios Liveris, Andrea C. Love, Gary P. Wormser, Ira Schwartz, Mary M. Petzke

**Affiliations:** 1 Department of Microbiology and Immunology, New York Medical College, Valhalla, New York, United States of America; 2 Division of Infectious Diseases, Department of Medicine, New York Medical College, Valhalla, New York, United States of America; University of Kentucky College of Medicine, United States of America

## Abstract

The capacity for *Borrelia burgdorferi* to cause disseminated infection in humans or mice is associated with the genotype of the infecting strain. The cytokine profiles elicited by *B. burgdorferi* clinical isolates of different genotype (ribosomal spacer type) groups were assessed in a human PBMC co-incubation model. RST1 isolates, which are more frequently associated with disseminated Lyme disease in humans and mice, induced significantly higher levels of IFN-α and IFN-λ1/IL29 relative to RST3 isolates, which are less frequently associated with disseminated infection. No differences in the protein concentrations of IFN-γ, IL-1β, IL-6, IL-8, IL-10 or TNF-α were observed between isolates of differing genotype. The ability of *B. burgdorferi* to induce type I and type III IFNs was completely dependent on the presence of linear plasmid (lp) 36. An lp36-deficient *B. burgdorferi* mutant adhered to, and was internalized by, PBMCs and specific dendritic cell (DC) subsets less efficiently than its isogenic B31 parent strain. The association defect with mDC1s and pDCs could be restored by complementation of the mutant with the complete lp36. The RST1 clinical isolates studied were found to contain a 2.5-kB region, located in the distal one-third of lp36, which was not present in any of the RST3 isolates tested. This divergent region of lp36 may encode one or more factors required for optimal spirochetal recognition and the production of type I and type III IFNs by human DCs, thus suggesting a potential role for DCs in the pathogenesis of *B. burgdorferi* infection.

## Introduction

*Borrelia burgdorferi*, a tick-transmitted spirochete, is the infectious agent of Lyme disease [1], [2]. This disease is a multisystemic disorder with possible neurologic, rheumatologic, and cardiac symptoms which develop following hematogenous dissemination of the bacterium from the skin to distal target tissues [3]. The potential for Lyme disease to develop from the bite of an infected tick is highly variable due to the existence in nature of distinct *B. burgdorferi* genotypes with diverse capacities to cause infection or to disseminate. The genome of *B. burgdorferi* type strain B31-MI consists of a single linear chromosome and 21 linear and circular plasmids [4], [5]. Linear plasmid 54 (lp54), circular plasmid 26 (cp26) and the chromosome are highly conserved and have been present in all tested natural isolates of *B. burgdorferi*. However, the presence and content of other plasmids vary among isolates [4]–[7]. A genotyping method for *B. burgdorferi* isolates based on restriction-fragment length polymorphism of the 16S–23S ribosomal DNA spacer region has been developed [8], [9]. A relationship between ribosomal spacer type (RST) and the capacity for disseminated infection was observed; RST1 strains are more frequently associated with disseminated infection in both Lyme disease patients and mice, whereas RST3 isolates are less frequently detected in the blood of patients and some do not disseminate in mice [10]–[13]. The molecular mechanisms underlying the differential risk for hematogenous dissemination of different *B. burgdorferi* genotypes, however, have not been elucidated.

*B. burgdorferi* induces the production of both pro- and anti-inflammatory cytokines through the nuclear factor-kappa B (NF-κB) signaling pathway [14]–[18]. Internalization of intact *B. burgdorferi* and subsequent degradation and release of pathogen associated molecular patterns (PAMPs) within the phagolysosome are critical events leading to full activation of the innate immune response to this extracellular pathogen [16], [17], [19]–[21]. Phagocytic uptake of intact spirochetes induces secretion of IFN-γ by NK cells and triggers both apoptosis and the production of high levels of pro-inflammatory cytokines by human monocytes [16], [17]. Recent findings have identified the endosomal receptors, TLR7 and TLR9 in dendritic cells, and TLR8 in monocytes, as pathogen recognition receptors (PRRs) essential for the production of type I IFN protein and the transcription of IFN-responsive genes by *B. burgdorferi*-stimulated human immune cells [20]–[23]. Moreover, Cervantes et al. provided evidence using human monocytes that coordinated interactions between TLR8 and TLR2 facilitate the development of a diverse pro- and anti-inflammatory cytokine response by human monocytes [21]. However, despite the contribution of TLR2 to the initiation of *B. burgdorferi*-triggered signaling cascades both at the cell surface and within phagolysosomes, TLR2 is not required for spirochetal uptake [19]. Hawley et al. recently identified CR3 (CD11b) as a MyD88-independent phagocytic receptor for *B. burgdorferi* that also modulates TNF production by murine bone marrow-derived macrophages [24]. The *B. burgdorferi* ligand for CR3, as well as spirochetal components that mediate internalization by other phagocytic cell populations, remain unknown.

Type I interferons (IFN-α/β), an innate defense classically associated with an antiviral immune state and produced at high levels by plasmacytoid dendritic cells (pDCs), are now known to be induced in response to a variety of intracellular and extracellular bacterial pathogens [20], [25]–[29]. Previously, using an ex vivo co-incubation model, we demonstrated that human pDCs and CD11c^+^CD14^+^ monocytoid cells produce IFN-α protein following phagocytosis of *B. burgdorferi* [20]. It has also been shown that IFN-α is present at higher levels in the serum of Lyme disease patients with multiple erythema migrans (EM) lesions, an indicator of disseminated infection, compared to those of patients with a single EM [26]. The expression of IFN-α in the EM lesion correlates with the presence of monocytoid (mDC) and plasmacytoid (pDC) dendritic cells, both of which are enriched approximately 5-fold in the EM lesion infiltrate as compared with the skin of uninfected controls [26]. The interactions of *B. burgdorferi* with a specific innate immune cell population might facilitate dissemination and promote Lyme disease pathogenesis.

The type III IFNs, or IFN-λs, discovered in 2003, are a family of novel cytokines that includes three members: IFN-λ1, IFN-λ2, and IFN-λ3, also known as IL-29, IL-28A, and IL-28B, respectively [30]. Similar to type I IFNs, IFN-λs are expressed primarily by monocytes, macrophages, and dendritic cells, and can be produced by these cells simultaneously with IFN-α and IFN-β [30]–[33]. Although initially identified as anti-viral proteins, recent studies demonstrate that type III IFNs are an important component of the innate immune response to non-viral pathogens, including *Salmonella enterica* serovar *typhimurium* [31], [34] and *Listeria monocytogenes* [35]. However, the expression pattern of the IFN-λ receptor (IFNλR) is far more restricted than that of the type I IFN receptor; IFNλR is expressed solely in epithelial tissues and epithelial-like cell types, suggesting that the effects exerted on innate immunity likely are more important for pathogens interacting with the epithelium [36], [37]. Notably, *L. monocytogenes* pathogenesis is promoted by type I IFNs but can be further enhanced by cooperative interactions between type I IFNs and IL-28B [35].

Two reports from our laboratory were the first to describe the induction of IFN-λ1 (IL-29) by *B. burgdorferi* [23], [38]. Love et al. demonstrated the production of IL-29 by TLR7-dependent signaling in human PBMCs in response to live *B. burgdorferi* or isolated *B. burgdorferi* RNA. Those results were generated using *B. burgdorferi* B515, an RST1 clinical isolate that disseminates in mice [11]. In the current study, the cytokine profiles induced by *B. burgdorferi* clinical isolates of varying genotypes were analyzed using a human PBMC ex vivo co-incubation model. Intriguingly, differential recognition by human dendritic cells was found to be dependent on a highly divergent region of lp36 which is conserved among RST1 isolates.

## Results

### *B. burgdorferi* Clinical Isolates Induce Type I and Type III IFNs in a Genotype-dependent Manner

**Figure 1:**
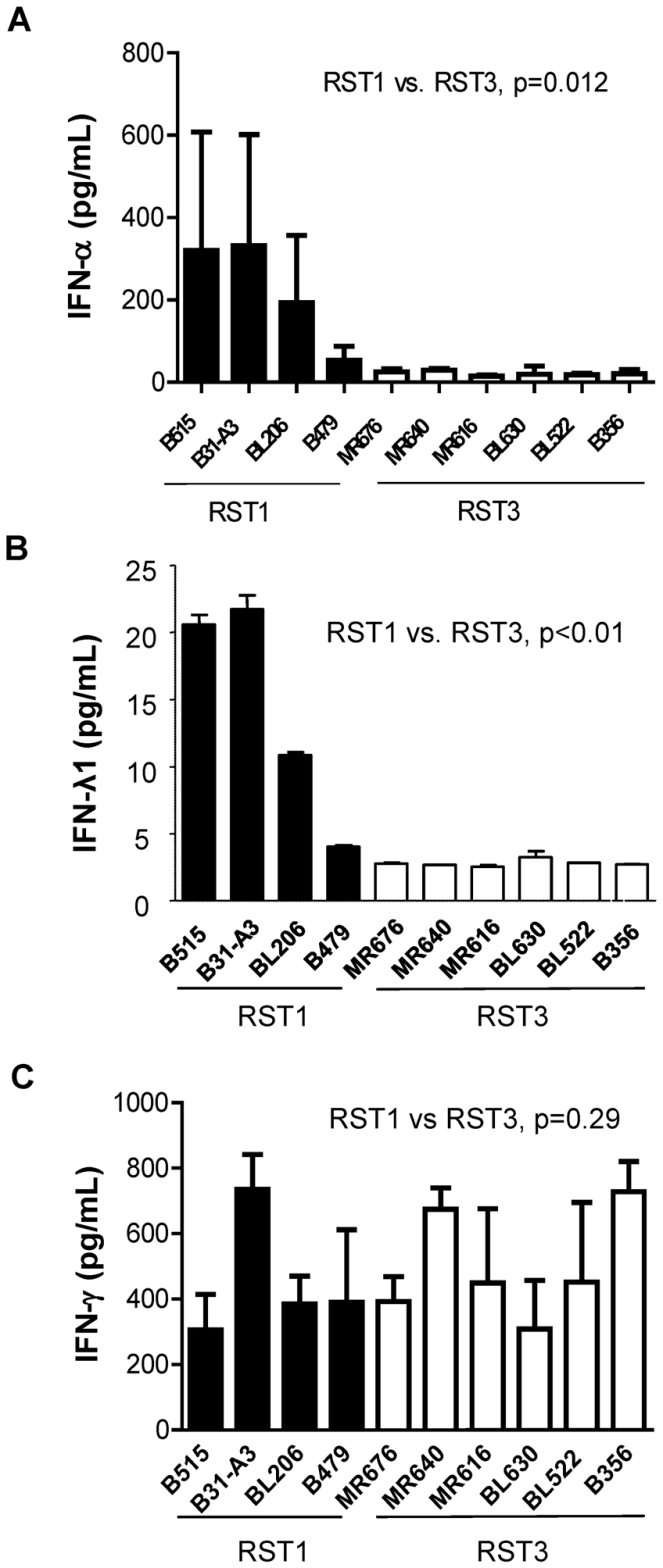
RST1 isolates induce expression of multiple interferons. Human PBMCs (4×10^6^) were co-incubated for 20 hrs with RST1 (black bars) or RST3 (white bars) *B. burgdorferi* clinical isolates (4×10^7^) (MOI = 10∶1). Concentrations of human IFN-α (***A***), IFN-λ1/IL29 (***B***), or IFN-γ (***C***) proteins in cell-free culture supernatants were quantitated by ELISA (***A*** *,* ***B***) or cytometric bead array (***C***). Columns represent the mean ± SD of values from three blood donors assessed in independent experiments, with the exception of (***B***) which represents results from a single donor. Statistical analysis was performed using a non-parametric, two-tailed Mann-Whitney U-test.

*B. burgdorferi* clinical isolates obtained from the blood or skin biopsies of early Lyme disease patients were classified into distinct genotypes [9]. The interferon response of human PBMCs to *B. burgdorferi* strains with varying genotypes was assessed using an ex vivo co-incubation model as previously described [20,20] (Table 1). IFN proteins in the cell-free culture supernatants were quantitated by ELISA (IFN-α, IFN-λ) or by cytometric bead array (IFN-γ). Representative results are shown in Figure 1. RST1 isolates induced production of 224.70±337.46 pg/mL of IFN-α, whereas RST3 isolates induced 21.57±14.86 pg/mL IFN-α (p = 0.012) (Fig. 1A). In addition, RST1 isolates were also found to elicit significantly higher production of IL-29/IFN-λ1, a type III IFN, as compared to RST3 isolates [RST1, 14.28±8.41 pg/mL; RST3, 2.78±0.24 pg/mL (p<0.01)] (Fig. 1B). In contrast, both RST1 and RST3 isolates induced equivalent amounts of IFN-γ (Fig. 1C). These results demonstrate that there is a significant correlation between the induction of IFN-α and IFN–λ1 and *B. burgdorferi* genotype.

**Table 1:** *B. burgdorferi* clinical isolates and strains used in this study.

Identifier	Genotype^a^	Biological source	Reference
B31-A3	RST1	B31 derivative	[101]
B31-4	RST1	B31 derivative	[39]
A3-M9 lp36−	RST1	B31 derivative	[43]
A3-M9 lp36−/lp36+	RST1	B31 derivative	[43]
B479	RST1	Human EM skin biopsy	[11]
B515	RST1	Human EM skin biopsy	[11]
BL206	RST1	Lyme disease patient blood	[10], [11]
B331	RST3A	Human EM skin biopsy	[11]
B356	RST3A	Human EM skin biopsy	[10], [11]
BL522	RST3A	Lyme disease patient blood	
BL630	RST3A	Lyme disease patient blood	
MR616	RST3A	Human EM skin biopsy	[7]
MR640	RST3A	Human EM skin biopsy	
MR676	RST3A	Human EM skin biopsy	

a Genotype was determined by PCR-RFLP analysis of 16S-23S ribosomal RNA gene spacer [9].

### *B. burgdorferi* Isolates of Varying Genotypes Induce Comparable Levels of Pro- and Anti-inflammatory Cytokines

In order to better understand the role that genotype plays in the *B. burgdorferi*-elicited cytokine response of human PBMCs, a human FlowCytomix bead array was employed to simultaneously measure various cytokine levels in cell-free culture supernatants from 20-hour co-incubations of PBMCs with *B. burgdorferi* isolates (Table 2). Protein levels for IL-1β, IL-6, IL-8 and TNF-α were the highest of the cytokines measured. Concentrations of IL-10 and IL-12p70 were markedly lower. IL-2, IL-4, IL-5 and TNF-β proteins were below the level of detection in most of the samples tested. No significant differences were detected when comparing RST1- and RST3-induced levels of any of the cytokines (Table 2). While it appeared that higher levels of IL-12p70 were induced by RST1 isolates relative to RST3 isolates, this difference did not achieve statistical significance (Table 2). In summary, of all cytokine proteins assayed, only IFN-α and IFN-λ1 levels differed significantly in PBMCs incubated in the presence of *B. burgdorferi* isolates of varying genotype, with higher induction by RST1 isolates.

**Table 2:** Pro- and anti-inflammatory cytokine production by RST1 and RST3 *B. burgdorferi* clinical isolates.

	Cytokine (pg/mL)					
Genotype	IL-1β	IL-6	IL-8	IL-10	IL-12p70	TNF-α
**RST1** * (n = 4)	24841±7187	79738±17430	73678±21296	9468±1876	3441±3243	45748±21388
**RST3** (n = 6)	21898±8148	71340±10956	69671±10190	9807±2641	2392±2728	43941±18476
**P value**	0.317	0.479	0.826	0.788	0.137	0.981

*B31-4 and A3-M9 lp36− were not included in the RST1 group when calculating averages and performing statistical analysis. Statistical significance was determined using a non-parametric Mann-Whitney U-test. Data shown are from three independent donors assessed in separate experiments.

### *B. burgdorferi* Mutants Lacking Linear Plasmid 36 (lp36) Elicit Virtually no IFN-α or IFN-λ1

**Figure 2:**
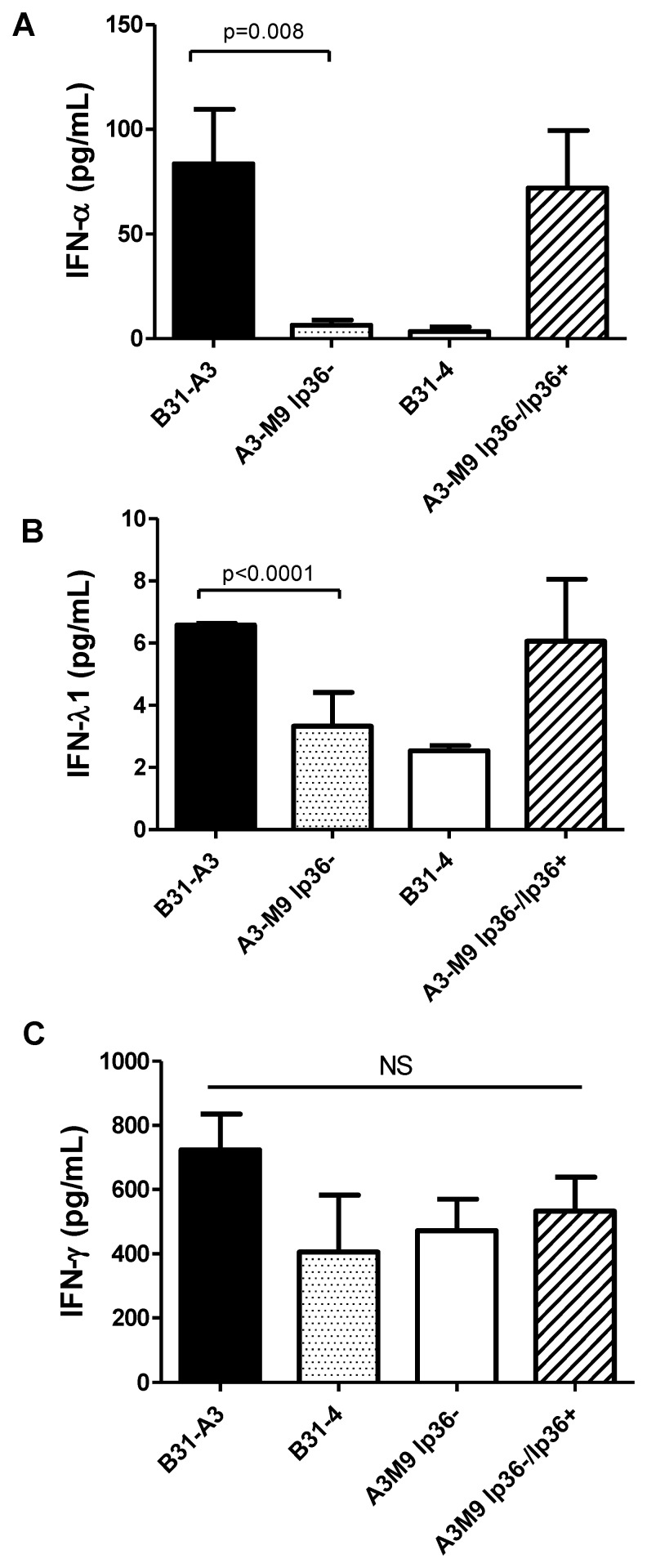
The presence of lp36 is required for IFN-α and IFN-λ induction. Human PBMCs (4×10^6^) were co-incubated for 20 hrs with 4×10^7^ wt B31-A3 (black bars), B31-4 (gray bars), A3-M9 lp36− (white bars) or A3-M9 lp36−/lp36+ (cross-hatched bar)(MOI = 10). Protein concentrations of human IFN-α (***A***), IFN-λ1 (***B***), or IFN-γ (***C***) in cell-free culture supernatants were quantitated by ELISA (***A***,***B***) or cytometric bead array (***C***). The results are shown as the mean ± SD of values from three to five blood donors assessed in two or three independent experiments. Statistics were obtained using a non-parametric Mann-Whitney U-test.

The sequenced *B. burgdorferi* genome of the *B. burgdorferi* type strain B31-MI consists of a single linear chromosome and 12 linear and 9 circular plasmids [4], [5]. B31 is an RST1 strain [9]. B31-4 is a derivative of strain B31 that lacks all linear plasmids [39]. This strain elicited approximately 3.46±4.17 pg/ml IFN-α protein, whereas B31-A3, a strain that contains all plasmids, elicited 95.91±58.91 pg/ml IFN-α (p = 0.02) (Figure 2A).

To identify the linear plasmid that may encode the factor(s) responsible for type I IFN induction, *B. burgdorferi* mutants that lacked single individual linear plasmids were assessed for their ability to induce IFN-α. These included B31 strains which lacked: lp28-1 [40], lp25 [40], two independent mutants lacking lp28-4 [41], [42], and two independent mutants lacking lp36 [43]. The absence of lp28-1, lp25, and lp28-4 had no effect on IFN-α production by human PBMCs (data not shown). However, the *B. burgdorferi* strains which lacked only lp36 elicited virtually no IFN-α protein. Results are shown in Figure 2A for A3-M9 lp36−, a B31 derivative that lacks lp36 but contains all other plasmids known to be required for infectivity [43]; an independently derived B31 strain lacking lp36 produced essentially identical results (data not shown). A3-M9 lp36− elicited only 4.93±4.95 pg/ml IFN-α protein (p = 0.008) (Figure 2A). Furthermore, strain A3-M9 lp36− complemented by the re-introduction of lp36 (designated as A3-M9 lp36−/lp36+) [43] produced IFN-α at levels equivalent to those induced by the parent strain B31-A3 (Figure 2A). *B. burgdorferi* strains B31-4 and A3-M9 lp36− also elicited significantly lower levels of IFN-λ1 as compared to B31-A3 (2.53±0.24 pg/mL and 2.97±1.25 pg/mL vs. 11.62±8.73 pg/mL, respectively; p<0.001 for both) (Figure 2B). In contrast, IFN-γ, IL-1β, IL-6, IL-8, IL-10, and TNF-α production by PBMCs was not dependent on lp36; B31-A3 and A3-M9 lp36− induced comparable levels of these cytokines (Figure 2C and Table 3). This finding suggests that a *B. burgdorferi* factor(s) required for induction of type I and type III IFN is likely encoded on lp36.

**Table 3:** Pro- and anti-inflammatory cytokine profiles elicited by *B. burgdorferi* B31-A3 and A3-M9 lp36−.

	Cytokine (pg/mL)					
Genotype	IL-1β	IL-6	IL-8	IL-10	IL-12p70	TNF-α
**B31-A3 (wt)**	21120±3259	112152±71306	54369±4144	8594±4851	916.9±842.6	6688±10922
**A3-M9 lp36**−	16535±9775	81210±56966	50509±12690	4475±1581	483.6±687.8	2973±4324
**P value**	0.151	0.421	0.691	0.095	0.222	0.486

Statistical analysis was performed using a non-parametric Mann Whitney U-test. Data shown are from three to five independent donors assessed in separate experiments.

### Strains B31-A3 and A3-M9 lp36− elicit Virtually Identical NF-κB-mediated Expression Profiles, but Differentially Induce Type I IFN Gene Transcripts

**Figure 3:**
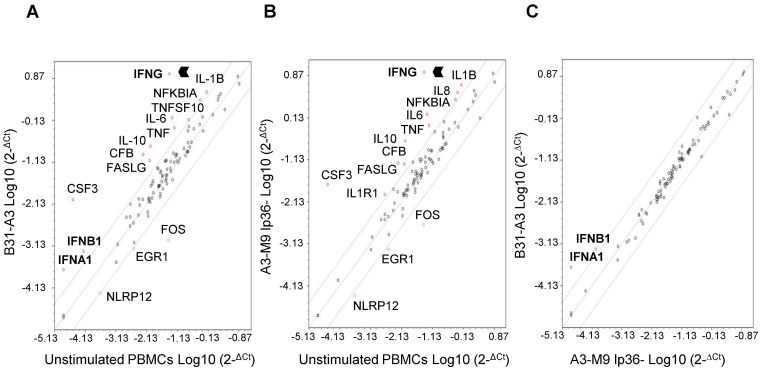
The absence of lp36 does not affect NF-κB-mediated signaling, but is required for transcription of *ifna1* and *ifnb1*. Human PBMCs (5×10^6^) were co-incubated for 12 hours with 5×10^7^ B31-A3 or A3-M9 lp36−. Equal amounts of PBMC total RNA from each of three biological replicates was pooled, converted to cDNA and used as a template for the Human NF-κB Signaling Pathway RT^2^ Profiler PCR Array. Genes that were transcriptionally induced or repressed ≥4-fold are indicated by red or green circles, respectively. Scatter plots depict transcriptional regulation in PBMCs stimulated with B31-A3 *(* ***A*** *)* or A3-M9 lp36− (***B***) versus unstimulated PBMCs. ***C*** *,* Comparison of transcript profiles of PBMCs stimulated with either B31-A3 or A3-M9 lp36− *B. burgdorferi*.

In order to obtain a global picture of the NF-κB response induced in human PBMCs exposed to B31-A3 or A3-M9 lp36−, PBMC total RNA was isolated following co-incubation and analyzed using an NF-κB RT^2^ Profiler PCR Array. This allows for the simultaneous quantitative measurement of 84 NF-κB-related gene transcripts. Similar transcriptional response profiles were generated by B31-A3 and A3-M9 lp36− (Figure 3). Genes which were commonly induced by either strain ≥4-fold relative to unstimulated PBMCs included those for IFN-γ, IL-10, TNF-α, IL-6, IL-1β, colony stimulating factor 3 (CSF3), complement factor B (CFB), Fas ligand (FASLG) and nuclear factor of kappa light polypeptide gene enhancer in B-cells inhibitor alpha (NFKBIA). Genes which were commonly repressed by either isolate ≥4-fold relative to unstimulated PBMCs consisted of those encoding NLR family pyrin domain containing 12 (NLRP12), early growth response 1 (EGR1) and FBJ murine osteosarcoma viral oncogene homolog (FOS) (Figure 3A and 3B, respectively). Notably, genes for the type I IFNs, IFNα1 and IFNβ1, were uniquely induced following PBMC stimulation with B31-A3 but not with A3-M9 lp36− (Figure 3A and 3B). A direct comparison of the expression profiles elicited by B31-A3 and A3-M9 lp36− revealed that these were the only two of the 84 genes on the NF-kB PCR array that were differentially regulated by the two strains (Figure 3C). These findings corroborate the IFN protein data shown in Figure 2 and further validate the importance of lp36 for induction of the type I IFN response.

### Spirochetes that Lack lp36 have Reduced Adherence to Human PBMCs and are Phagocytosed Less Efficiently

**Figure 4:**
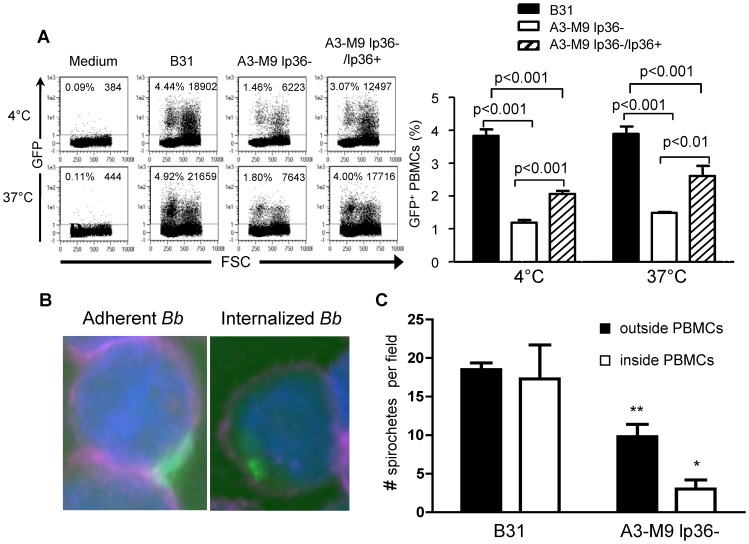
The adhesion and uptake of *B. burgdorferi* is significantly reduced when lp36 is absent. Human PBMCs (4×10^6^) were co-incubated with 4×10^7^ GFP-tagged B31-A3 (black bars), A3-M9 lp36− (white bars) or A3-M9 lp36−/lp36+ (cross-hatched bars) for 6 hours at 4°C or 37°C. ***A***, Flow cytometry was performed to compare the ability of the GFP-tagged *B. burgdorferi* strains to associate with PBMCs. Representative dot plots are shown on the left. Graphs (right) depict the mean percentage ± SD of PBMCs identified as FITC+, indicating either adhesion (4°C) or adhesion and uptake (37°C) of GFP-tagged *B. burgdorferi*. Data are representative of results from three blood donors assessed in triplicate. A one-way ANOVA with a Tukey’s post-test was used to determine statistical significance. ***B*** and ***C***, Three-dimensional fluorescence deconvolution microscopy was performed to quantify spirochetes adherent to, or internalized by, PBMCs. Samples were co-incubated with GFP-tagged *B. burgdorferi* for 6 hours at 37°C. PBMC outer cell membranes were labeled with APC-conjugated anti-CD45 to label the outer cell membrane (cyan) and DAPI (blue) was used to label the cell nucleus. Single optical sections of images acquired under 100X magnification were examined for adherent (***B***, left) or internalized (***B***, right) GFP-tagged *B. burgdorferi*. ***C***, Columns show the mean (± SD) number of spirochetes outside (black bars) or inside (white bars) PBMCs. Data were collected from single optical sections taken from five 100X fields from each of three independent biological replicates. An unpaired, two-tailed Student’s *t*-test was used for statistical analysis.

The production of IFN-α by *B. burgdorferi*-stimulated human PBMCs is dependent on phagocytosis of the bacterium, as it is blocked by the addition of cytochalasin D [20]. This suggested the possibility that lp36-deficient *B. burgdorferi* failed to induce IFN-α as a result of a defect in uptake of the mutant by phagocytic cells. To test this hypothesis, B31, A3-M9 lp36− and A3-M9 lp36−/lp36+ transformants containing green fluorescent protein (GFP) were produced and their association with PBMCs was evaluated by flow cytometry or microscopy. To verify that introduction of the GFP reporter plasmid did not affect the ability to elicit type I IFN, the GFP-transformed strains were assayed for IFN-α induction in human PBMCs and were found not to differ significantly from the non-GFP-transformed strains (data not shown). Adhesion and association of GFP-tagged B31, A3-M9 lp36− and A3-M9 lp36−/lp36+ that had been co-incubated with human PBMCs for 6 hr at either 4°C (prevents phagocytosis) or 37°C (allows phagocytosis) were assessed by flow cytometry. A significantly higher percentage of B31 adhered to PBMCs (3.9±0.2%GFP+ PBMCs vs. 1.50±0.04%, p<0.001; 4°C), and associated with PBMCs (3.88±0.23% vs. 1.48±0.04%GFP+ PBMCs, p<0.001; 37°C), than did A3-M9 lp36− (Figure 4A). A partial restoration in adhesion and association was observed with the lp36-complemented *B. burgdorferi* strain (Figure 4A).

The same co-incubation conditions were used to acquire samples for 3D-fluorescence deconvolution microscopy (optical sectioning) to further differentiate adherent from internalized spirochetes. Cytocentrifugation was used to deposit cells from co-incubations onto slides. Cells were fixed and stained with anti-CD45, a pan-leukocyte marker, which labels the PBMC outer membrane and allows the identification of spirochetes inside PBMCs, as opposed to spirochetes adherent to the outside of the cell membrane. Five 100X fields from three separate co-incubations (B31 or A3-M9 lp36−) at 37°C were analyzed. Figure 4B shows a representative single optical section from a deconvolved Z-stack for an adherent GFP-tagged B31 (left panel) versus an internalized B31 *B. burgdorferi* (right panel). As shown in Figure 4C, significant more wild type *B. burgdorferi* per field were adherent to the outside of PBMCs as compared to lp36− mutants (18.5±0.87 vs. 9.8±1.6 A3-M9 lp36−; p<0.01). Similarly, significantly more wild type spirochetes were internalized relative to lp36− *B. burgdorferi* (17.3±4.4 vs. 3.0±1.2; p<0.05) (Figure 4C). These data demonstrate that the absence of lp36 results in defects in both *B. burgdorferi* spirochetal adhesion to, and internalization by, PBMCs.

### The Association of *B. burgdorferi* with mDC1s and pDCs is Significantly Reduced when lp36 is Absent

**Figure 5:**
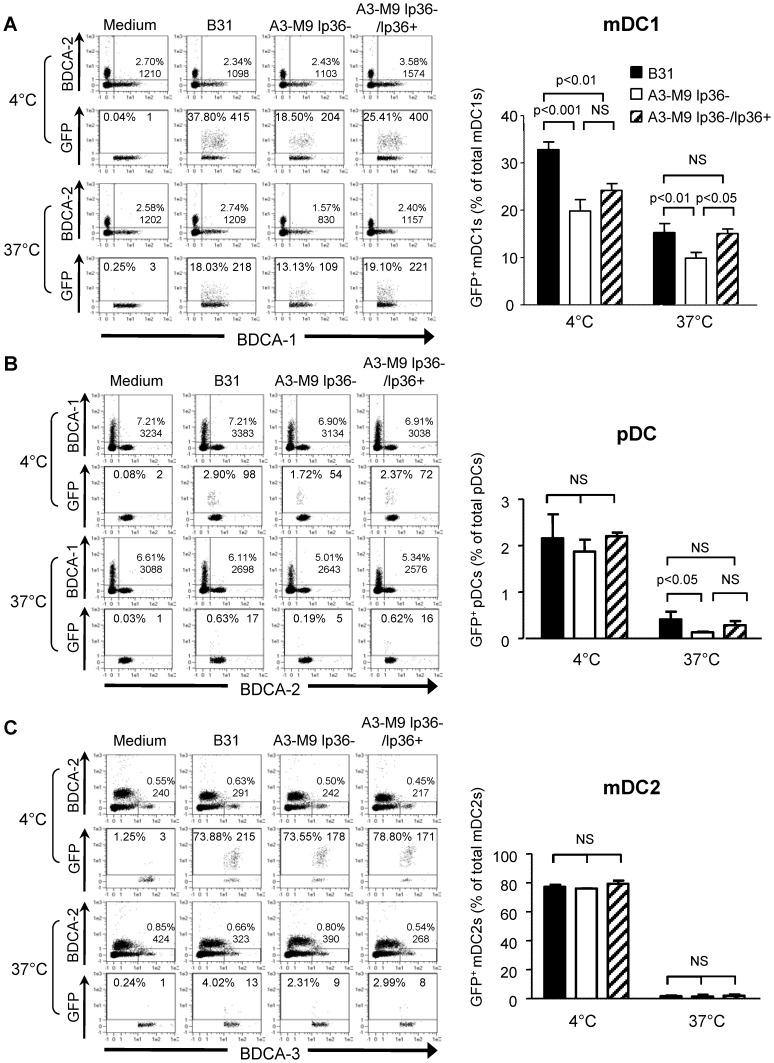
lp36 contributes to the association of *B. burgdorferi* with specific populations of dendritic cells. Human PBMCs (4×10^6^) were co-incubated with 4×10^7^ GFP-tagged B31 (black bars), A3-M9 lp36− (white bars) or A3-M9 lp36−/lp36+ (cross-hatched bars) *B. burgdorferi* for 6 hours at 4°C or 37°C. The percentages of GFP^+^ mDC1s (CD19^−^CD3^−^BDCA2^−^BDCA1^+^) (***A***), pDCs (CD19^−^CD3^−^BDCA2^+^BDCA1^−^) or (***B***) mDC2s (CD19^−^CD3^−^BDCA3^+^BDCA2^−^) (***C***) were determined by multiparameter flow cytometry. Dot plots representing 500,000 collected events are provided to illustrate gating strategies (left). Column graphs represent the mean and standard deviation of three biological replicates (right). Statistical analysis was performed using a one-way ANOVA with a Tukey’s post-test for multiple comparisons.

PBMCs are a heterogeneous population of innate immune cells consisting of T-cells, B- cells, monocytes, NK cells and dendritic cells [44]. Human blood dendritic cells are classified as plasmacytoid dendritic cells (pDCs), myeloid BDCA1^+^ dendritic cells (mDC1s) and myeloid BDCA3^+^ dendritic cells (mDC2s) [45]. Although pDCs constitute only ∼0.4% of the total PBMC population, they specialize in the production of large amounts of type I IFNs [46], [47]. Previous findings from this laboratory demonstrated that pDCs and CD11c^+^CD14^+^ monocytoid cells are the primary producers of IFN-α on co-incubation with *B. burgdorferi* [20]. The cell population(s) involved in the differential association and phagocytosis of *B. burgdorferi* based on the presence of lp36 was further examined using flow cytometry (Figure 5). A significantly higher percentage of BDCA1^+^ mDC1s (15.3±1.9 vs. 9.9±1.2; p<0.01) and BDCA2^+^ pDCs (0.4±0.2 vs. 0.1±0.02; p<0.05) were associated with GFP-tagged B31 *B. burgdorferi* than with the lp36− strain. Re-introduction of lp36 restored spirochetal association with mDC1s and pDCs to levels observed with the wild type (Figure 5A and 5B, respectively). However, it should be noted that the percentages of pDCs associated with lp36− mutant or complemented strains at 37°C were not significantly different. Interestingly, both wild type and lp36−deficient *B. burgdorferi* strongly adhered to approximately 80% of BDCA3^+^ mDC2s at 4°C, but failed to associate with these cells at 37°C (Figure 5C), although the total numbers of mDC2 cells remained unchanged (Figure 5C, left). Together, these results demonstrate that lp36 is required for optimal recognition and phagocytosis of spirochetes by pDC and mDC1 populations.

### RST1 Isolates contain a Region of lp36 which is Absent in RST3A Isolates

**Figure 6:**
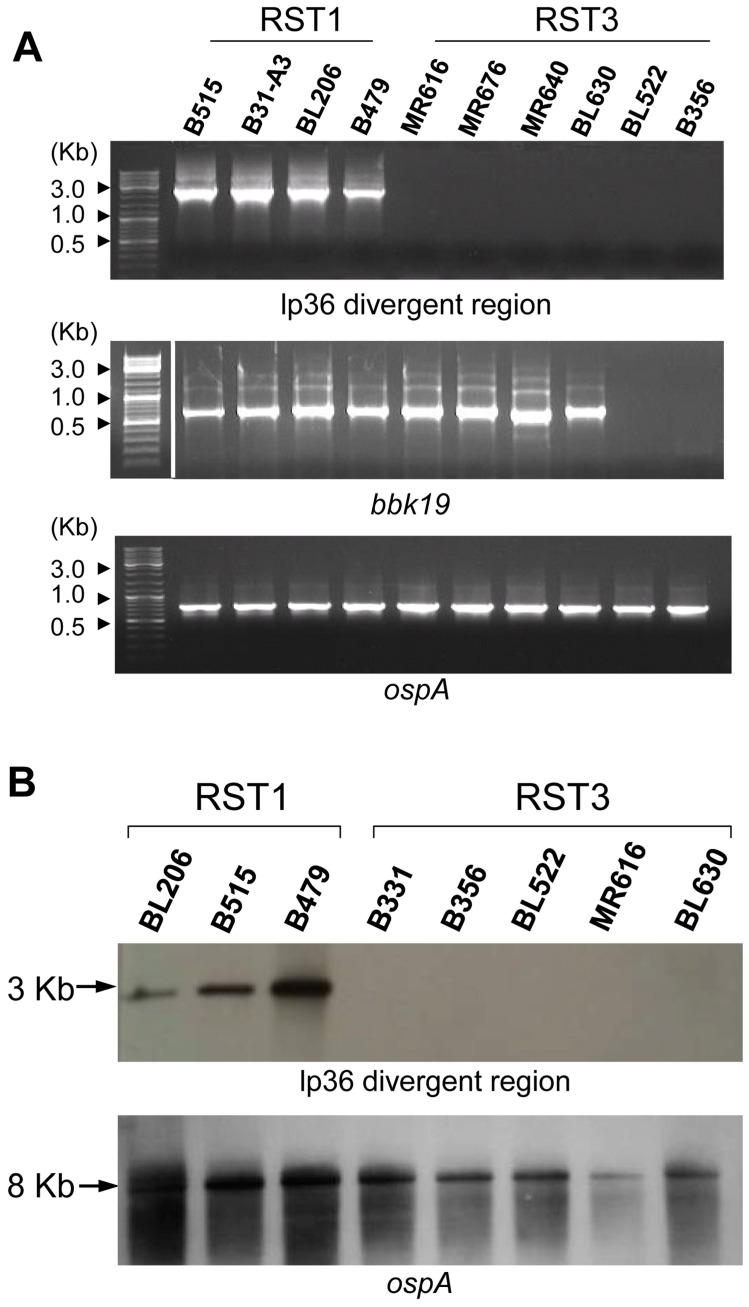
RST1 isolates contain a region of lp36 that is lacking in RST3 isolates. RST1 and RST3 *B. burgdorferi* isolates were grown to late log phase prior to isolation of plasmid DNA. (***A***) PCR primers were designed to amplify a ∼2.5 kilobase region on the distal end of lp36. The presence of lp36 was assessed by amplification of *bbk*19, an ORF located adjacent to the plasmid partition locus. PCR products were analyzed by gel electrophoresis in 1% agarose gels prepared in 1X TBE buffer and stained with ethidium bromide. (**B**) Southern blotting of *Spe*I digests of total genomic DNAs of *B. burgdorferi* isolates was performed using a probe specific for the variable region of lp36 (upper panel). As a control, membranes were stripped and re-probed for *B. burgdorferi ospA* (BBA15), located on lp54 (lower panel).

It has been previously observed that plasmids constitute the most divergent portion of the *B. burgdorferi* genome and that lp36 is highly variable among clinical isolates of *B. burgdorferi*: the divergent region of lp36 was shown to encompass ORFs BBK35-BBK50 [7]. It was, therefore, of interest to explore whether the presence or absence of this region of lp36 correlated with the differential induction of IFN-α described earlier (Figure 1). PCR primers (primer set lp36A; Table 4) were designed to amplify a 2.5-kb sequence (*bbk42*-*bbk45*) within the divergent region of lp36. The same four RST1 isolates and six RST3 isolates that were tested for IFN-α production (Figure 1) were scored for the presence of the lp36-divergent sequence. All RST1 isolates yielded a PCR product of expected size, whereas no PCR product was obtained for RST3 isolates (Figure 6A, top panel). The presence of *bbk19*, which is located in a conserved area of lp36 adjacent to the plasmid partition locus, was assessed to determine if the RST3 isolates lack the entire plasmid. All RST1 and four of the six RST3 isolates tested were PCR positive for *bbk19*; a *bbk19* PCR product was not detected for B356 or BL522 (Figure 6A, middle panel). B356 was previously shown to lack lp36 [7]; the absence of *bbk*19 in BL522 suggests that this isolate may also lack lp36. All isolates yielded a PCR product for *ospA*, which is located on the conserved lp54 plasmid [7], [48] (Figure 6A, bottom panel).

**Table 4:** Oligonucleotides used for plasmid content analysis.

Primer name	Primer sequence	Predicted fragment size (bp)	Plasmid nucleotide coordinates
lp36A-Forward	5′-GGG AAA TGC TAT AAG CCT CAA GCC CAG CC-3′	2480	26244–28724
lp36A-Reverse	5′-GCT TAG CCT GCA CCA AGC TAT TGC TAA GG-3′		
lp36B-Forward	5′-GCATCTCCTATAAGTATAC-3′	184	27069–27253
lp36B-Reverse	5′-TCCCTGGTAAAGTAATTCATTTATCC-3′		
BBK19-Forward	5′-CTA TTG TTT GTC CAA GTC TTA ATC-3′	1196	12269–13465
BBK19-Reverse	5′-ATT GTA CGA AGT TGA ATT TTA GCA TC-3′		
OspA-Forward	5′-GGG AAT AGG TCT AAT ATT AGC C-3′	770	9410–10160
OspA-Reverse	5′-TTT CAA CTG CTG ACC CCT C-3′		

One limitation of PCR is the requirement that primer and target DNA contain nucleotide sequences that are nearly 100% complementary; even single nucleotide substitutions in the primer binding sequence can substantially reduce primer annealing and subsequent PCR product yield. Therefore, Southern hybridization of *Spe*I-digested genomic DNA was performed for eight clinical isolates using a gene probe specific for a 184-bp sequence located within the 2.5-kB lp36 divergent region. These included three RST1 and four RST3 isolates that had been assessed by PCR. Due to insufficient yields of DNA, RST3 isolates MR676 and MR640 could not be included; B331, an RST3 clinical isolate that does not disseminate in mice [11], was substituted. The predicted *Spe*I digestion fragment size of 3085 bp (lp36 nucleotide coordinates 24828–27913) corresponding to the lp36 variable region was observed in all three of the RST1 isolates but none of the RST3 isolates, confirming the PCR results (Figure 6B). These findings suggest that the RST3 isolates studied possess an ‘lp36-like’ plasmid that lacks the divergent region conserved among RST1 isolates, and that this region of lp36 encodes the factor(s) required for *B. burgdorferi*-elicited type I IFN production by human dendritic cells.

## Discussion

In this study, we used an established human PBMC ex vivo co-incubation model [20] to explore the effect of *B. burgdorferi* genotype on the induction of cytokines, including three major classes of IFNs, and to delineate the host cell-pathogen interactions that contribute to the differential induction of IFNs. We observed that RST1 clinical isolates induced significantly higher levels of IFN-α and IFN-λ1 (IL-29) than did RST3 clinical isolates. In contrast, there were no significant differences between these two *B. burgdorferi* genotypes with regard to levels of induced IFN-γ, IL-1β, IL-6, IL-8, IL-10, IL-12p70 and TNF-α. Strle and colleagues reported that *B. burgdorferi* RST1 isolates, OspC type A genotype in particular, are associated with greater inflammation and more severe disease [49]. After a 24-hour co-incubation of human PBMCs with *B. burgdorferi* clinical isolates in that study, significantly higher amounts of IL-6, IL-8, CCL3, and CCL4 were produced in response to RST1 isolates as compared to those incubated with RST2 or RST3 isolates. In addition, RST1 isolates induced significantly higher levels of IFN-α than did RST2 isolates [49]. There are several differences between the experiments performed by Strle et al. and those described here that may contribute to the contrasting results with regard to IL-6 and IL-8 production. Strle et al. used an MOI of 25∶1 and co-incubation of 24 hours or 5 days, whereas an MOI of 10∶1 and co-incubation for 20 hours were employed in this study. Importantly, despite some differences, both studies demonstrate that there is a correlation between *B. burgdorferi* genotype, the capacity to cause a disseminated infection and the ability to induce a type I IFN response.

The finding that the capacity of *B. burgdorferi* to induce a type I and type III IFN response is genotype-dependent, and correlates with pathogenic potential, raises a number of questions. One important inquiry relates to the genomic component of *B. burgdorferi* responsible for the differential IFN induction. The genome of *B. burgdorferi* is comprised of a single linear chromosome (∼910 kb) and twelve linear and nine circular plasmids which total an additional 610 kb [4], [5]. There have been extensive studies correlating plasmid content and the pathogenesis of *B. burgdorferi* infection [40], [41], [43], [50]–[53]. To identify the *B. burgdorferi*-encoded factor(s) required for the induction of type I and type III IFNs, various *B. burgdorferi* mutants lacking specific linear plasmids were screened for their ability to induce a type I IFN response. Strikingly, *B. burgdorferi* strains that selectively lacked only lp36 had a significantly attenuated capacity to induce the production of type I and type III IFNs. In contrast, both wild-type and lp36-deficient *B. burgdorferi* induced comparable levels of IL-1β, -6, -8, -10, IL12-p70, IFN-γ and TNF-α. In support of these data, out of 84 different NF-κB-related genes, only the type I IFN gene transcripts *ifna1* and *ifnb1* were found to be induced by wild-type B31-A3 relative to A3-M9 lp36−.

The lp36 of *B. burgdorferi* type strain B31-MI is a 36-kilobase plasmid that encodes 54 putative ORFs, many of which are annotated as encoding hypothetical proteins [4]. There is a limited body of literature regarding lp36 and its encoded ORFs. Using whole cell DNA from a group of 15 North American *B. burgdorferi* isolates, Palmer et al. determined that lp36-derived DNA probes hybridized to targets of significantly different size in B31 as compared to B31-MI [6]. Terekhova and colleagues performed comparative genome hybridization and Southern blot analysis with various clinical isolates of *B. burgdorferi* and determined that RST1 isolates contain an lp36 plasmid virtually identical to that of B31-MI, whereas the non-RST1 isolates contain an “lp36-like” plasmid that is considerably different from the lp36 of B31-MI, yet contains the lp36 partition locus [7]. Jewett et al. investigated the role of lp36 in the infectious cycle of *B. burgdorferi* and showed that while lp36 was not required for spirochete survival in the tick, *B. burgdorferi* lacking lp36 exhibited an approximately 4-log increase in ID_50_ relative to wild-type *B. burgdorferi* in a murine infection model [43]. The lower infectivity was partially attributed to the loss of *bbk17* (*adeC*), which encodes an adenine deaminase [43]. In addition to *bbk17*, the functions of several other lp36-encoded genes have been examined: *bbk32* encodes a fibronectin-binding protein that may promote full infectivity in mice [54]–[56], and *bbk07* has been investigated as a potential marker for serodiagnosis of Lyme disease [57], [58]. We tested mutant strains lacking *bbk07*, *bbk17* or *bbk32* and found that these mutants induced IFN-α at levels that were indistinguishable from those elicited by the wild-type strain (data not shown). It is worthy to note that none of these three genes is located within the divergent region (*bbk35*-*bbk50*) of lp36.

The current investigation was designed to explore the relationship between *B. burgdorferi* genotype and induction of a type I IFN response. Rather than a systematic analysis of all OspC genotypes, the study focused on RST1 (OspC type A) genotypes that have a high probability of causing disseminated infection and RST3 strains with OspC genotypes (E, G, M and U) that have a low probability of causing disseminated infection [59]. A limitation of the study is that several RST2 and RST3 genotypes (OspC types H, K and I) that have a high likelihood of producing disseminated infection in humans were not analyzed. It would be of interest in a future investigation to explore whether these *B. burgdorferi* genotypes induce high levels of type I IFN by human PBMCs. Recently, whole-genome sequences for other *B. burgdorferi* isolates have become available [60] and provide further evidence for a correlation between genotype, disseminating capacity and lp36 content. Strain N40, a *B. burgdorferi* OspC type E tick isolate that disseminates in mice [61], contains sequences that are highly homologous to the variable region of B31 lp36. However, the genetic information is divided between lp17 and lp36: the last ∼1400 nucleotides of the 2.5 kb fragment are present on N40 lp17 whereas the first ∼800 nucleotides of the fragment remain on N40 lp36 [60]. In contrast, *B. burgdorferi* isolates 118a (OspC type J), 72a (OspC type G) and 94a (OspC type U) [62], [63], which based on OspC type are likely to be RST3 isolates, do not encode the 2.5-kb variable region of B31 lp36. Taken together, these data suggest that this highly variable region may encode a factor(s) that is responsible for the induction of type I and type III IFNs by human PBMCs and that may contribute to the differential pathogenic potential of RST1 and RST3 *B. burgdorferi* isolates.

We have previously demonstrated that *B. burgdorferi* induces production of type I IFNs by human PBMCs in a TLR7- and TLR9-dependent manner [20]. While generally host protective in viral infections, the biological effects of type I IFNs on the outcome of non-viral infections can be variable. A potent type I IFN response contributes to the control of infection by some bacteria [64]–[73] but promotes the pathogenesis of other bacteria, including *Listeria monocytogenes* [25], [74], *Mycobacterium bovis* [75], *Mycobacterium tuberculosis* [76] and uropathogenic *E. coli* (UPEC) [77]. In the context of *B. burgdorferi* infection, type I IFN signaling has been shown to be crucial for the development of arthritis in a susceptible mouse model [78], [79]. In addition, significantly higher levels of serum IFN-α have been detected in human patients with multiple EM lesions, a symptom of disseminated *B. burgdorferi* infection, as compared to patients with a single EM [26]. Similar to type I IFNs, IFN-λs are expressed primarily by monocytes, macrophages and dendritic cells and can be produced by these cells simultaneously with IFN-α and IFN-β [30]–[33]. Type I and type III IFNs possess overlapping biological activities: both can induce MHC class I antigen expression, regulate transcription of a common subset of interferon-stimulated genes in various cell lines [30], increase NK and T cell cytotoxicity [80], promote Th1 responses [81] and mediate cellular apoptosis [82]. Recent studies demonstrate that type III IFNs are an important component of the innate immune response to non-viral pathogens, particularly those that are associated with epithelial tissues, including *Salmonella enterica* serovar *typhimurium* [31], [34] and *L. monocytogenes* [35]. We have previously shown that IFN-λ1 production can be elicited from human PBMCs by a disseminating isolate of *B. burgdorferi*, as well as by purified RNA from this same isolate [23], [23,38,38].

We have previously established that phagocytosis is required for *B. burgdorferi*-induced IFN-α production by pDCs and mDC precursors [20]. The finding that lp36 is required for the induction of both type I and type III IFN suggests that lp36 may mediate the association of *B. burgdorferi* with DC populations by encoding a phagocytic ligand. This hypothesis is supported by multi-parameter flow cytometry and 3-D fluorescence deconvolution microscopy. *B. burgdorferi* that lacked lp36 were attenuated not only in the ability to adhere to human PBMCs, but also in promoting phagocytic uptake by these cells. The absence of lp36 did not affect the ability of *B. burgdorferi* to either adhere to or associate with mDC2s; it is therefore unlikely that this cell type has a critical role in mediating spirochete dissemination. In contrast, optimal association of *B. burgdorferi* with mDC1s and pDCs was found to be dependent on the presence of lp36; thus these particular cells, while comprising an extremely small percentage (∼1%) of total PBMCs, may preferentially associate with *B. burgdorferi* and aid the spirochetes in hematogenous dissemination.

The data reported here support a conclusion that appears to be paradoxical: *B. burgdorferi* with greater capacity for dissemination are preferentially phagocytosed by DCs. However, a body of literature has emerged that describes a crucial role for DCs in immune suppression. Tolerogenic DCs have been shown to promote the generation of regulatory T-cells (Tregs) through expression of the immunosuppressive enzyme 2,3-indoleamine dioxygenase (IDO) [83]–[86]. IDO expression has been implicated in the generation of both central and peripheral tolerance, but a particularly crucial function appears to be the establishment of localized immune privilege in epithelial tissues. IDO-expressing DCs and Foxp3^+^ Tregs have been implicated in the development of various infectious and autoimmune pathological conditions of the epithelium, including lichen planus, lupus vulgaris, sarcoidosis and infections caused by *Mycobacterium leprae*, *Haemophilus ducreyi*, UPEC and *Leishmania* sp. [77], [87]–[91]. Of particular relevance to the current study, Tregs have been found in the peripheral blood of patients with secondary syphilis, a disease that evolves following initial skin infection with *Treponema pallidum*, another spirochetal pathogen [92]. Although it is known that human monocytoid and plasmacytoid DCs are present in the EM lesion infiltrate of Lyme disease patients [26], [93], [94], the potential role of these innate immune cells in determining disease outcome remains to be established. We hypothesize that recognition of *B. burgdorferi* by DCs may direct the differentiation of these cells towards a tolerogenic phenotype, resulting in localized immune suppression that may be exploited by *B. burgdorferi* to establish infection and enable dissemination. These questions are the topics of ongoing investigation in our laboratory.

In summary, this study demonstrates that *B. burgdorferi* lp36 is indispensable for the induction of IFN-α and IFN-λ1 by human PBMCs and for full recognition of *B. burgdorferi* by human dendritic cell populations. In contrast, *B. burgdorferi* genotype did not affect induction of IFN-γ or other NF-κB-dependent cytokines. The ability of *B. burgdorferi* to induce IFN-α and IFN-λ was associated with the presence of a variable 2.5-kb segment of lp36 (*bbk42–bbk45*) that was conserved among the RST1 isolates, but absent in all RST3 isolates evaluated. These results provide new insight into the connection between *B. burgdorferi* genotype, lp36 content and pathogenic potential. Although the presence of DCs in EM lesions has been reported, the potential role of these innate immune cells in determining disease outcome remains to be defined. The ability of tolerogenic DCs to generate localized immune suppression suggests a mechanism that may be exploited by *B. burgdorferi* in order to establish infection and enable subsequent dissemination.

## Materials and Methods

### Ethics Statement

Written informed consent was obtained from all human subjects in accordance with protocols approved by the Institutional Review Board of New York Medical College.

### Culture of *Borrelia burgdorferi*

Low-passage *B. burgdorferi* clinical isolates were obtained from the blood or skin biopsies of the EM lesions of early Lyme disease patients enrolled in a prospective study at the Lyme Disease Diagnostic Center at New York Medical College, Valhalla, NY [12], [95]. B31-4, a derivative of *B. burgdorferi* strain B31 that lacks all linear plasmids, has been described [39]. A3-M9 lp36− and A3-M9 lp36−/lp36+, both B31 derivative strains, have been previously described [42]. Spirochetes were cultured in modified Barbour-Stoenner-Kelly (BSK-S) medium [96]. To simulate the temperature shifts that occur during tick-to-mammal transmission and that may enhance the expression of virulence factors, spirochetes were grown at 25°C, subcultured and incubated at 37°C until cultures reached the mid- to late-log phase of growth (4 to 8×10^7^/ml). Spirochetes were enumerated and assessed for motility by dark-field microscopy [95]. The genotypes and biological sources of the *B. burgdorferi* clinical isolates for which data are reported in this study are provided in Table 1.

### Isolation of Human PBMCs

Venous blood was obtained from five healthy adult volunteers who had no prior history of Lyme disease, had not been vaccinated for Lyme disease and were seronegative for *B. burgdorferi* antibodies as confirmed by Western immunoblotting. Written informed consent was obtained from all subjects before blood collection, in accordance with the protocol approved by the Institutional Review Board of New York Medical College. Blood was collected directly into BD-Vacutainer CPT tubes (BD Biosciences) and PBMCs were isolated by centrifugation according to the manufacturer’s instructions. PBMCs were washed twice with Hank’s Balanced Salt Solution (HBSS) and resuspended in RPMI 1640 medium without phenol red containing 10% (v/v) heat-inactivated and endotoxin-free fetal bovine serum (FBS) (Hyclone). The viability of PBMCs was determined by trypan blue staining and was found to be >90%.

### Co-incubation of PBMCs with *B. burgdorferi*

Ex vivo stimulation of freshly isolated PBMCs by *B. burgdorferi* was performed essentially as previously described [20]. Spirochetes were centrifuged at 8000×g for 10 min, washed twice with HBSS and resuspended in RPMI 1640 without phenol red containing 10% (v/v) heat-inactivated and endotoxin-free FBS (Hyclone). Live *B. burgdorferi* were added to 4×10^6^ (or 5×10^6^ for experiments involving RNA isolation) viable PBMCs at a multiplicity of infection (MOI) of 10∶1 (spirochetes:PBMC) in 24-well tissue culture plates, in a final volume of 1.1 ml. Co-cultures were maintained in a humidified 37°C incubator containing 5% CO_2_ for the specified time period.

### Measurement of Secreted Cytokine Proteins

PBMCs were co-incubated with *B. burgdorferi* for 20 hrs. Cell-free culture supernatants were prepared by removal of PBMCs at 300×g for 10 min followed by centrifugation of the supernatant at 9000×g for 10 min to remove non-adherent spirochetes and cellular debris. Cell-free supernatants were aliquoted and stored at −20°C until further use. Concentrations of human IFN-α and IFN–λ1 were quantitated using the Human Verikine IFN-α (PBL Biomedical Laboratories) or the Human IL-29 ELISA Ready-Set Go! Kit (eBioscience), respectively. Protein levels of IFN-γ, IL-1β, IL-2, IL-4, IL-5, IL-6, IL-8, IL-10, IL-12 p70, TNF-α and TNF-β in cell-free culture supernatants were measured using the FlowCytomix Human Th1/Th2 11plex Kit (BMS810FF;Bender MedSystems) according to the manufacturer’s instructions. Data were acquired with a MACSQuant analyzer (Miltenyi Biotec) and analyzed using FlowCytomix Pro Software (Bender MedSystems).

### RNA Isolation

PBMCs were co-incubated with *B. burgdorferi* as described above for 12 hours. The contents of each well were transferred to a 1.5-ml microcentrifuge tube and centrifuged at 300×g for 5 min at 4°C. PBMCs were washed with HBSS and RNA was isolated using the RNeasy Plus Mini Kit with QIAshredder and gDNA eliminator columns (Qiagen). RNA was eluted in 20 µl of RNase/DNase-free water and stored at −80°C after the addition of 0.8 µl of RNase inhibitor (40 U/µl; Promega).

### NF-κB-mediated Gene Expression PCR Array

PBMCs were co-incubated in triplicate with *B. burgdorferi* for 12 hr and RNA was isolated as described above. The RT^2^ First Strand Kit (SABiosciences) was used for cDNA synthesis, according to the manufacturer’s instructions. cDNA from biological triplicates was pooled and used as a template for the RT^2^ Profiler PCR Array Human NFκB Signaling Pathway (PAHS-025A;SABiosciences), according to the manufacturer’s instructions. This array contains primer pairs for 84 well-characterized NF-κB-related genes, 5 housekeeping genes and various quality controls in a 96-well plate format. Transcript levels were quantified based on the 2^−ΔΔCT^ method [97] through the measurement of SYBR green fluorescence using the RT^2^ Real-Time SYBR Green/Rox PCR Master Mix (PA-012; SABiosciences).

### Generation of Green Fluorescent Protein-expressing *B. burgdorferi*

A GFP reporter plasmid, based on plasmid pCE320 [17], [98], was used to electrotransform *B. burgdorferi* A3-M9 lp36− *B. burgdorferi* as previously described [99]. This plasmid contains the GFP structural gene fused to the constitutive *flaB* promoter of *B. burgdorferi* and encodes for gentamicin resistance. Transformants were selected by growth in BSK medium containing 40 µg/mL of gentamicin and plated by limiting dilution to obtain single clones. The GFP-expressing lp36 complement, A3-M9 lp36−/lp36+, was constructed using the GFP reporter plasmid pSPC-S [100]. This plasmid contains the GFP structural gene fused to the constitutive *flaB* promoter of *B. burgdorferi* and encodes for streptomycin resistance. Transformants were selected by growth in BSK-S medium containing 100 µg/mL streptomycin. Wild-type B31 *B. burgdorferi* expressing GFP was kindly provided by Drs. Justin Radolf and Melissa Caimano, University of Connecticut Health Center [98].

### Multi-parameter Flow Cytometric Analysis

Human PBMCs were co-incubated for 6 hours at 4°C or 37°C with GFP-tagged *B. burgdorferi* at MOI of 10. Following co-incubation, the contents of each well were transferred to a 5 mL FACS tube and pelleted at 300×g for 10 min. Cells were washed twice with 1 mL HBSS to remove unattached spirochetes, fixed with 3.2% formaldehyde in HBSS for 30 min, pelleted and resuspended in 0.5 mL FACS stain buffer (1X PBS, 0.2% bovine serum albumin, pH 7.4). Total numbers of GFP+ PBMCs were determined by flow cytometric analysis of 100,000 to 300,000 events using a MACSQuant Analyzer and MACSQuantify software (Miltenyi Biotec).

Dendritic cell populations present within PBMCs were identified by labeling of surface antigens. Following co-incubation, the contents of each well were transferred to two, 5 mL FACS tubes (∼2×10^6^ cells each) and centrifuged at 300×g for 10 min. Cells were washed once with 1 ml HBSS and once with 1 ml FACS stain buffer. To block Fc receptors, cells were resuspended in 70 µl stain buffer containing 1 µg/mL human IgG1, kappa, purified myeloma protein (Sigma 5154). 30 µL of the appropriate antibody staining panel (mDC1/pDC or mDC2) was then added to each sample. Antibody fluorochrome conjugates consisted of anti-CD19-eFluor450 (mouse IgG1,k, clone HIB19 eBioscience 48-0199), anti-CD3-eFluor450 (mouse IgG1,k, clone UCHTI eBioscience 48-0038), anti-BDCA1 (CD1c)-APC (mouse IgG2a, clone AD5-8E7 Miltenyi 130-090-903), anti-BDCA2 (CD303)-PE (mouse IgG1, clone AC144 Miltenyi 130-090-511) and anti-BDCA3 (CD141)-APC (mouse IgG1, clone AD5-14H12 Miltenyi 130-090-907). Cell populations were defined as follows: mDC1s (CD19^−^CD3^−^BDCA2^−^BDCA1^+^), pDCs (CD19^−^CD3^−^BDCA2^+^BDCA1^−^) or mDC2s (CD19^−^CD3^−^BDCA3^+^BDCA2^−^). Samples were mixed well, covered and refrigerated at 4–8°C for 10 min. Cells were washed by adding 2 mL stain buffer followed by centrifugation at 300×g for 10 min at 4°C. Cells were then fixed in 800 µL of 3.2% formaldehyde in HBSS, covered and stored overnight at 4–8°C. Following fixation, cells were pelleted at 300×g for 10 min at 4–8°C and resuspended in 500 µL stain buffer. A minimum of 500,000 events per sample were collected on a MACSQuant analyzer and analyzed using MACSQuantify software (Miltenyi).

### Quantitation of Internalized versus Extracellular Spirochetes using 3-D-fluorescence Deconvolution Microscopy

Human PBMCs were co-incubated with GFP-tagged *B. burgdorferi* for 6 hours at 4°C or 37°C. Cells were pelleted at 300×g for 5 minutes, washed twice with tissue culture grade 1X PBS to remove non-adherent spirochetes and deposited onto glass microscope slides by cytocentrifugation. Cells were fixed in 3.7% formaldehyde for 20 minutes and stored in 1X PBS overnight at 4°C. Cells were protected from light and stained for 1 hour at room temperature with APC-conjugated mouse anti-CD45 (cyan) (Invitrogen; MHCD4505), a pan-leukocyte cell membrane marker, to define the PBMC outer membrane. Coverslips were mounted using Vectashield containing DAPI (Vector Laboratories, Inc.). Counts were obtained from deconvolved images using an inverse filter algorithm. Five 100X fields (∼250 total PBMCs) from each of three independent co-incubations were examined in a blinded manner for adherent or internalized spirochetes. Samples were imaged on a Zeiss Axiovert 200 fluorescence microscope and data were analyzed using Axiovision 4.6.3 software (both from Carl Zeiss, Inc.).

### PCR Analysis of *B. burgdorferi* Linear Plasmid Sequences

Plasmid DNA was isolated from 4-ml *B. burgdorferi* cultures (1×10^7^ to 1×10^8^ cells/ml) with the Qiaprep Spin Miniprep kit (Qiagen) and resuspended in 30 µl of nuclease-free water. PCR primers (Table 4) were designed to amplify a 2.5-kB sequence on lp36, encompassing *bbk42*–*bbk45* [7], [62]. Each PCR mixture contained approximately 10 ng DNA, 250 µM deoxynucleoside triphosphates (Roche), 10 ng of each primer and 1 U *Taq* DNA polymerase (Denville Scientific, Inc.). PCR conditions were as follows: initial denaturation at 95°C for 3 minutes; 35 cycles of 95°C for 1 minute, 60°C for 40 seconds, 72°C for 3 minutes; and final extension at 72°C for 2 minutes. PCR of *bbk19* was performed to assess the presence or absence of lp36. PCR parameters were as follows: initial denaturation at 95°C for 3 minutes, followed by 35 cycles of 95°C for 30 seconds, 48°C for 30 seconds, 72°C for 3 minutes, and final extension at 72°C for 2 minutes. *ospA* (*bba15*) was amplified as a control for the presence of plasmid DNA using the following conditions: initial denaturation at 95°C for 3 minutes; followed by 35 cycles of 95°C for 30 seconds, 52°C for 30 seconds, 72°C for 3 minutes; and final extension at 72°C for 2 minutes. PCR products were analyzed by electrophoresis in 1% agarose gels prepared in 1X TBE buffer and stained with ethidium bromide.

### Southern Hybridization

Total DNA was isolated from 10-ml cultures of *B. burgdorferi* B31-MI using a commercial DNA extraction kit (DNeasy Blood and Tissue Kit; Qiagen) and suspended in 100 µl nuclease-free water. DNA (3 µg) was digested overnight at 37°C with *Spe*I (10 U) in 50 µL 1X Buffer 2 (New England Biolabs) supplemented with 100 µg/ml BSA. Digested DNA was separated by electrophoresis on a 1% agarose gel prepared in 1X TBE buffer.

Probes to *ospA* and lp36 were separately generated by PCR using 10 ng B31-MI DNA template and 10 ng of each primer (Table 4). PCR parameters for *bba15* (*ospA*) were as previously reported [48]. A 184-bp sequence located in the divergent region of lp36 was amplified using the lp36-B primer set and the following thermocycler conditions: initial denaturation at 95°C for 2 minutes; 35 cycles of 94°C for 30 seconds, 42°C for 30 seconds and 72°C for 30 seconds; and final extension at 72°C for 2 minutes. PCR products were purified using a commercial kit (Qiagen) and labeled with digoxygenin-11-dUTP according to the manufacturer’s protocol (Roche Molecular Biochemicals). Southern blotting was performed as previously described [48]. Hybridization was first performed for the lp36 sequence. Hybridization patterns were visualized by addition of CDP-Star chemiluminescent substrate (Sigma-Aldrich) followed by exposure of the membrane to radiographic film for 2 to 4 minutes. The membrane was then stripped and re-probed for *ospA*.

### Statistical Analysis

Statistical significance was determined by one-way Analysis of Variance (ANOVA), a non-parametric Mann-Whitney U-test, a Kruskal-Wallis test, or a two-tailed, unpaired Student’s *t*-test using GraphPad Prism 5 software (GraphPad Software, Inc.), as appropriate.
